# Underappreciated layers of antibody-mediated immune synapse architecture and dynamics

**DOI:** 10.1128/mbio.01900-24

**Published:** 2024-12-11

**Authors:** Benjamin S. Goldberg, Margaret E. Ackerman

**Affiliations:** 1 Thayer School of Engineering, Dartmouth College, Hanover, New Hampshire, USA; 2 Geisel School of Medicine, Dartmouth College, Hanover, New Hampshire, USA; The Ohio State University, Columbus, Ohio, USA; Icahn School of Medicine at Mount Sinai, New York, New York, USA

**Keywords:** antibody, effector function, immune synapse, mechanism of action

## Abstract

The biologic activities of antibody drugs are dictated by structure-function relationships—emerging from the kind, composition, and degree of interactions with a target antigen and with soluble and cellular antibody receptors of the innate immune system. These activities are canonically understood to be both modular: antigen recognition is driven by the heterodimeric antigen-binding fragment, and innate immune recruitment by the homodimeric constant/crystallizable fragment. The model that treats these domains with a high degree of independence has served the field well but is not without limitations. Here, we consider how new insights, particularly from structural studies, complicate the model of neat biophysical separation between these domains and shape our understanding of antibody effector functions. The emerging model endeavors to explain the phenotypic impact of both antibody intrinsic characteristics and extrinsic features—fitting them within a spatiotemporal paradigm that better accounts for observed antibody activities. In this review, we will use insights from recent models of classical complement complexes and T cell immune synapse formation to explore how structural differences in antibody-mediated immune synapses may relate to their functional diversity.

## INTRODUCTION

**Fig 1:**
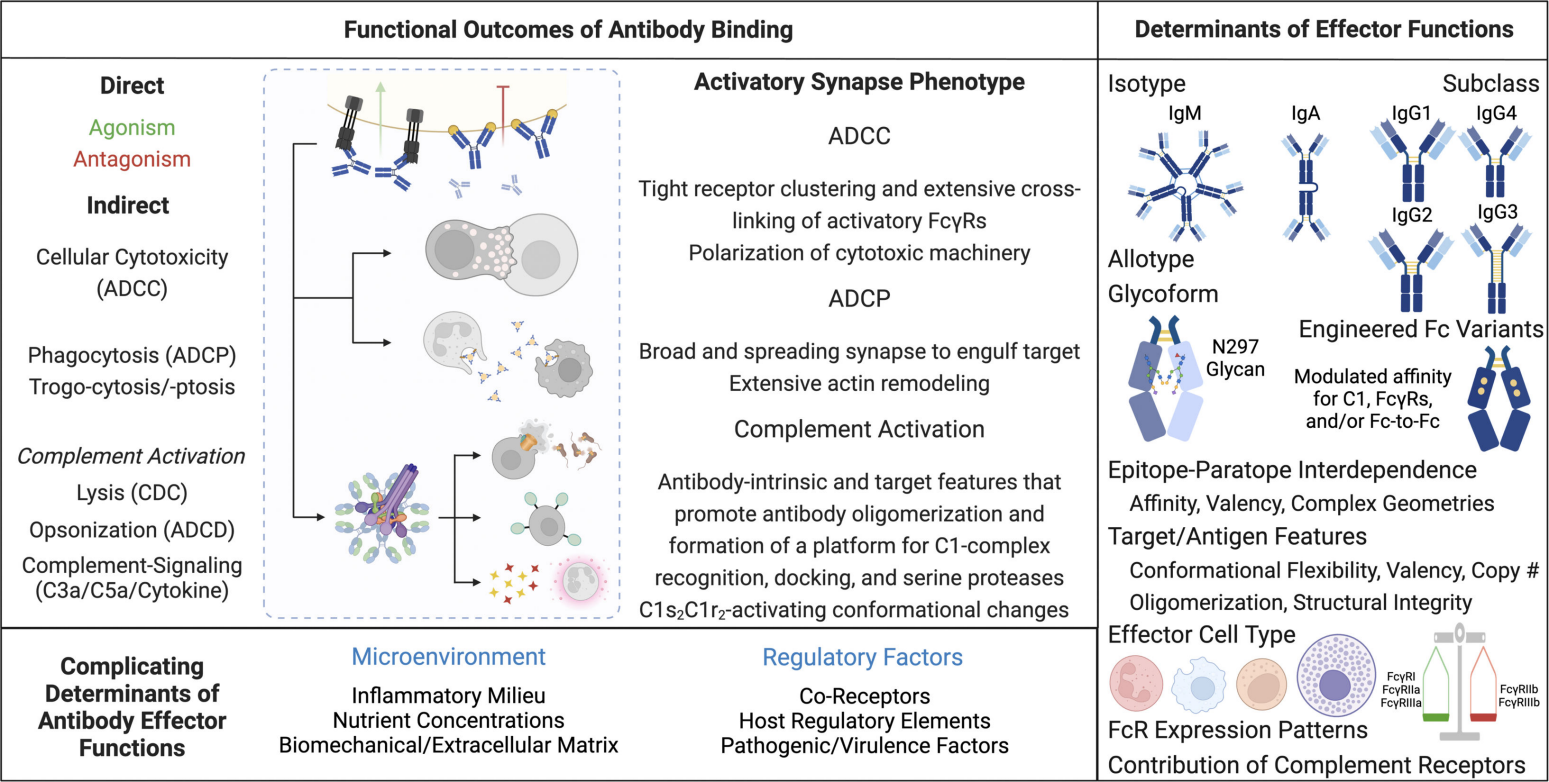
Determinants of antibody immune synapse architecture and dynamics. A broad range of effector, antibody, and target characteristics underlie antibody functional profiles. Antibodies can mediate a diverse set of immunologically and pathologically relevant innate effector functions. The kind(s) and degree to which an antibody elicits these activities is dependent on a number of factors. Given antibody recognition of its target, factors that immediately constrain these activities are the identity, frequency, and status of effectors present in the local tissue, and the biophysical characteristics of the antibody molecule. The former is shaped by host genetics and disease status shaping the microenvironmental milieu. These factors influence the types and expression levels of FcRs on recruited effector cells. Given fully optimal 1-to-1 interactions between a monoclonal antibody and its target antigen, the cell-surface densities of each and their ability to diffuse laterally within the membrane are critical components of signaling and activation. Such factors exist on a continuum and explain some but not all of the functional heterogeneity observed within groups of comparable antibodies. ADCP, antibody-dependent cellular phagocytosis; ADNP, antibody-dependent neutrophil phagocytosis; ADCC, antibody-dependent cellular cytotoxicity; CDR, complementary determining region; FcR, Fc receptor.

Antibodies are a dominant and growing class of biopharmaceutical agents based on their specificity and potency. Evidence from fields ranging from infectious disease to neoplastic disorders has linked antibody-mediated effector function (AMEF) to *in vivo* outcomes (1–18). These studies provide strong evidence motivating development and use of strategies to manipulate the ability of mAbs to direct the potent clearance activities of innate effector mechanisms. Such strategies have generally focused on optimizing interactions between the Fc domain and downstream effectors, but it is now well-established that a multitude of antibody, target, and effector parameters integrate to influence the degree of AMEF (Fig. 1). Better understanding of precisely how these factors influence diverse activities would improve modeling and design efforts (19–24), and reduce reliance on empirical pre-clinical evaluation and lead selection as it exists today. To date, the inability to comprehensively account for the numerous factors influencing AMEF *a priori* has necessitated analysis on an antibody-by-antibody basis in a range of imperfect assays and animal models.

Because the behavior of biological systems is fundamentally dictated by structure-function relationships, the clinical activity of monoclonal antibody (mAb) interventions emerges from the kind, composition, and degree of interactions with their target antigen and with antibody receptors of the innate immune system. These activities are typically considered to be modular and both structurally and functionally bifurcated: antigen recognition is driven by the heterodimeric antigen-binding fragment (Fab), and innate immune recruitment by the homodimeric constant/crystallizable fragment (Fc). In this model, beyond the ability of antigen recognition to drive direct activity, the Fc domains of adjacent antibodies become locally concentrated on the surface of a multivalent target particle or as part of a higher-order antibody-antigen immune complex (IC) and elicit the functions of the innate immune system via self-interactions and those with complement proteins and diverse Fc receptors (FcRs). The model that treats Fab and Fc domains with a high degree of independence, and in which local antibody concentration drives effector function has served the field well but is not without its limitations.

This simplified model is incomplete as evidenced by recent studies demonstrating higher-order or non-canonical interactions that regulate the efficiency of effector functions. For example, significant evidence indicates that the ability of adjacent surface-bound IgG Fc fragments to oligomerize results in more efficient complement activation (25, 26). Others have suggested a direct role of the Fab in FcγR-antibody interactions (27–29). It is now widely understood that mAb function can be highly context-dependent. In other words, although any antibody of the IgG1 subclass is theoretically able to engage with host immune effector mechanisms, this is hardly borne out empirically. For example, Bruel et al. assessed antigen binding levels and complement activation by a panel of HIV-1 specific antibodies that recognize a range of conserved regions on HIV-1 envelope glycoprotein (Env). They found that the level of antibody binding to surface-expressed Env on a transduced cell line alone did not correspond to the mAbs’ ability to elicit antibody-dependent complement deposition (C3b; ADCD) or antibody-dependent complement-mediated lysis (ADCML), nor was activity consistent within mAbs grouped by target region (30). Similar observations have been made in the setting of mAbs targeting cancer-associated antigens (31, 32) and for receptor-agonizing mAbs (33, 34). As a result, even when other intrinsic antibody characteristics are more or less equal, AMEF differs – in some cases, dramatically – between antibodies that recognize the same antigen or even epitope. Functional differences among relatively similar antibodies have been reported for other cellular effector functions as well: for example, antibody-dependent cellular cytotoxicity (ADCC) mediated by natural killer (NK) cells against HIV-infected target cells demonstrated significant ranges of activity across and within mAbs grouped by epitope specificity (35). For ADCC and other AMEF that rely upon cellular effectors, processes occur at cell-cell interfaces and involve higher-order architectures and more complex dynamics and topological constraints.

In this way, antibody-mediated synapses may well resemble other immune synapses for which spatial and temporal factors have been better integrated. Seminal work on the canonical T cell synapse illustrates how spatial constraints and temporal factors can impact cell-cell interactions in immunity. Currently, less is known at this level of detail for antibody-mediated synapses, including how antibody-based differences are linked to different intracellular signaling networks and ultimately to heterogeneous effector activities. Here, we highlight observations and lessons that have emerged linking intrinsic antibody features and spatial factors to regulation of effector cell activity.

## IMMUNE SYNAPSE (IS)

**Fig 2:**
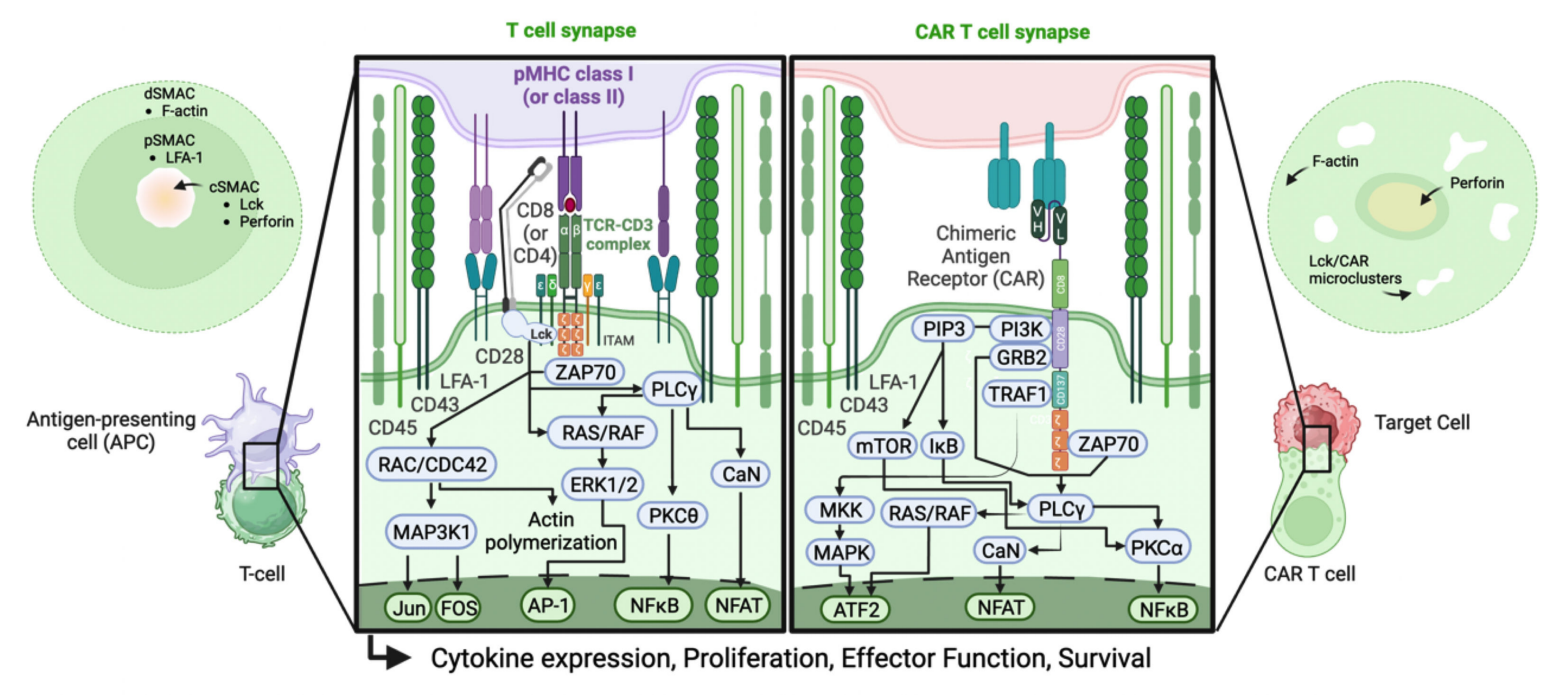
T cell immune synapse composition and signaling. The classical T cell immune synapse with central and peripheral regions and molecules that sort by size is pictured at the left and compared with the synapse structure observed for CAR T cells at the right. Signal cascades, shown in the center, differ in association with differences in the spatial organization of the synapse. Created with Biorender.

The term “immunological synapse” was coined in 1999 to describe the elaborate adhesion complex between T cells and antigen-presenting cells (APCs) that regulate antigen-specific activation of T cells (36). While the structured interface between another cytolytic immune population, NK cells, and their targets was described and reported at the same time (37), the T cell synapse remains the most well-characterized and has been described and reviewed elsewhere (38, 39). The exquisite sensitivity, elaborate spatial organization, and specificity of T cell activation is well-defined (Fig. 2). The formation of cell-cell contacts for communication (40) or subsequent high-stakes decision-making is foundational to many immune processes, which may bear similar layers of structural, mechanical, and temporal complexity. Even within T cell biology, ISs that are anatomically and functionally distinct from the canonical IS have been characterized (41). Insights gleaned from the study of both canonical and atypical ISs may be readily applicable to less well-studied settings such as ISs controlling AMEFs.

The canonical T cell synapse provides a solid foundation to understand how the structural features of individual molecules at the cell-cell synapse can interact (Fig. 2). The multiple membrane components present at the synapse itself, also known as the supramolecular activation cluster (SMAC), mediate processes involved in recognition, junction formation, stabilization, activation, and release. These components have been shown to exist in microclusters (42, 43) that condense and segregate into a pattern of three concentric rings that can be most readily described as a bulls-eye. The central SMAC (cSMAC) consists of APC major histocompatibility complex (MHC)-displaying antigenic peptides that are in complex with specificity-encoding cognate T cell receptors (TCRs). As the cSMAC forms via central migration of MHCp–TCR complexes, T cell integrin LFA-1 in complex with APC surface glycoproteins migrate to surround the cSMAC, eventually forming the adhesive peripheral SMAC (pSMAC). Distally, the so-named dSMAC forms the outermost ring and contains synapse-stabilizing proteins size-excluded from the more central areas. A consequence of the role of this IS structure in stabilizing the cSMAC, even single, low-affinity antigen-specific MHCp-TCR complexes can drive robust T cell activation (44, 45).

At the scale of cell-cell binding, the number of noncovalent protein-protein interactions sums substantially, and thus biomechanical forces are hypothesized to be involved in sensing (46), activation (47), and release. In fact, the interplay between topology and biomechanical forces is an exciting area of investigation that supplies another link between protein structure and cellular activity (48). Understanding of the spatial characteristics of the TCR-CD3 complex assemblies (49) is aiding the investigation of competing theories regarding mechanisms of TCR activation (50), the majority of which suggests that biomechanical forces are instrumental to activation. Analogously, tensile forces have been implicated in NK cell IS formation (51), suggesting a more general role for such typically overlooked factors in the context of other synapses.

### Lessons from engineered T cells

Understanding differences between immune synapses formed by natural TCRs and T cells redirected by chimeric antigen receptors (CARs), or by bispecific T cell engagers may assist in the development of high-throughput screening or methodologies or eventually rational design approaches. To compare TCR- and CAR-mediated IS structure, signaling, and function, Davenport et al*.* used a cell line co-expressing a natural TCR and a second-generation CAR specific for different targets. The authors found that CAR-Ts had a faster off rate than TCR-Ts, which correlated with more rapid pSMAC and dSMAC signaling, faster lytic granule migration to the synapse, and faster detachment from the target (52). The CAR synapse in this study was anatomically nonclassical and functionally more potent compared with the TCR-T synapse. These CAR ISs appeared disrupted, were less reliant on or even lacked a distinct adhesion ring, formed a multifocal pattern with Lck microclusters, and built aberrant lytic foci due to differential actin polymerization (Fig. 2).

The observation that redirected cytotoxic lymphocytes display heterogeneous functional activity linked to anatomical differences in the IS (53) has led to the notion that the IS could be used as a design metric for standardization or an *a priori* predictor of the safety and efficacy of engineered T cells or T cell-engaging biologics (54, 55). Redirected myeloid (56, 57) and NK cells (58) are also being pursued as intervention strategies, and characterization of their ISs may provide high-resolution characterization of their cell-cell synapses, signaling programs, and associated functional outcomes.

Relevant to AMEF, each effector cell type and relevant antibody receptor may be expected to exhibit their own sets of structure-function relationships in dictating the fate of opsonized targets. Comparison of the anatomy of other- and antibody-based ISs holds promise in broadly defining how extracellular stimuli shape intracellular signaling in effector cells and the fate of the targets thereof. Filling this knowledge gap will depend on building the linkages between extracellular factors and the dynamic behavior of effector cells.

### Lessons from non-traditional antibody-mediated immune synapses

Antibody-mediated immune cell redirection, for example, by bridging target and effector cell with synthetic bispecific antibodies, is another promising strategy to recruit specific effector mechanisms or even to engage immune checkpoint inhibitors synchronously (59–61). Factors that influence Ag-Ab complex formation, such as affinity for target antigen, were shown in an early study to improve cytotoxic function of bispecific antibody (bsAb)-redirected NK cells (62), and the critical importance of membrane mobility on forming cross-linked complexes at cell-cell junctions is highlighted in quantitative models (63). In this system, spatial factors influencing both Ag–Ab complex formation – Ag size (64–66), epitope location (64, 67), and Ab format (68) – and immune synapse activation – e.g., cytoskeletal motility and membrane fluidity (69), and segregation of activating receptors at close focal contacts (43, 70, 71) combine to regulate activation. Differences between effector cell systems, including the identities and states of intracellular signaling domains and pathways, translate to distinct activation sensitivities, which ultimately necessitate empirical assessment. Together, the features that regulate non-traditional antibody-mediated ISs are suggestive of those involved in AMEF such as ADCC and ADCP.

### Lessons from complement

Antibody-mediated complement activation has been implicated in protective and pathological contexts alike (72). Upon recognition of an antibody-opsonized target, it is believed C1q undergoes a conformational change that activates its associated serine protease dimers, C1s2C1r2, which exposes the catalytic domains of C1s that subsequently cleave its C4 substrate to C4b, and then C2 to C2b to form the C3 convertase (C4bC2b) (73). The requirement of avid engagement by the hexameric C1 complex increases the probability that activation of the complement pathway will exclusively occur in response to ICs formed in the context of regularly repeated molecular patterns, such as oligomeric membrane proteins or components of bacterial cell walls that promote the formation of higher-order complexes, for which antibody Fcs can reach critical threshold densities.

For complement recruitment, a high degree of Ag and epitope dependence has been long understood (74–76). Target specificity has been linked via Ag size, density, and membrane fluidity to the ability to form higher-order complexes upon target antigen cross-linking. The distinction between type I and type II α-CD20 mAbs is, in part, defined by their mode of binding linked to differential primary mechanisms of target killing (32). For example, type I (e.g., rituximab) and type II (e.g., obinutuzumab) α-CD20 mAbs recognizing overlapping epitopes can display categorically distinct biological properties, while type I α-CD20 mAbs targeting different epitopes altogether (e.g., rituximab and ofatumumab) can elicit a similar range of activities with varying degrees of potency (77).

**Fig 3:**
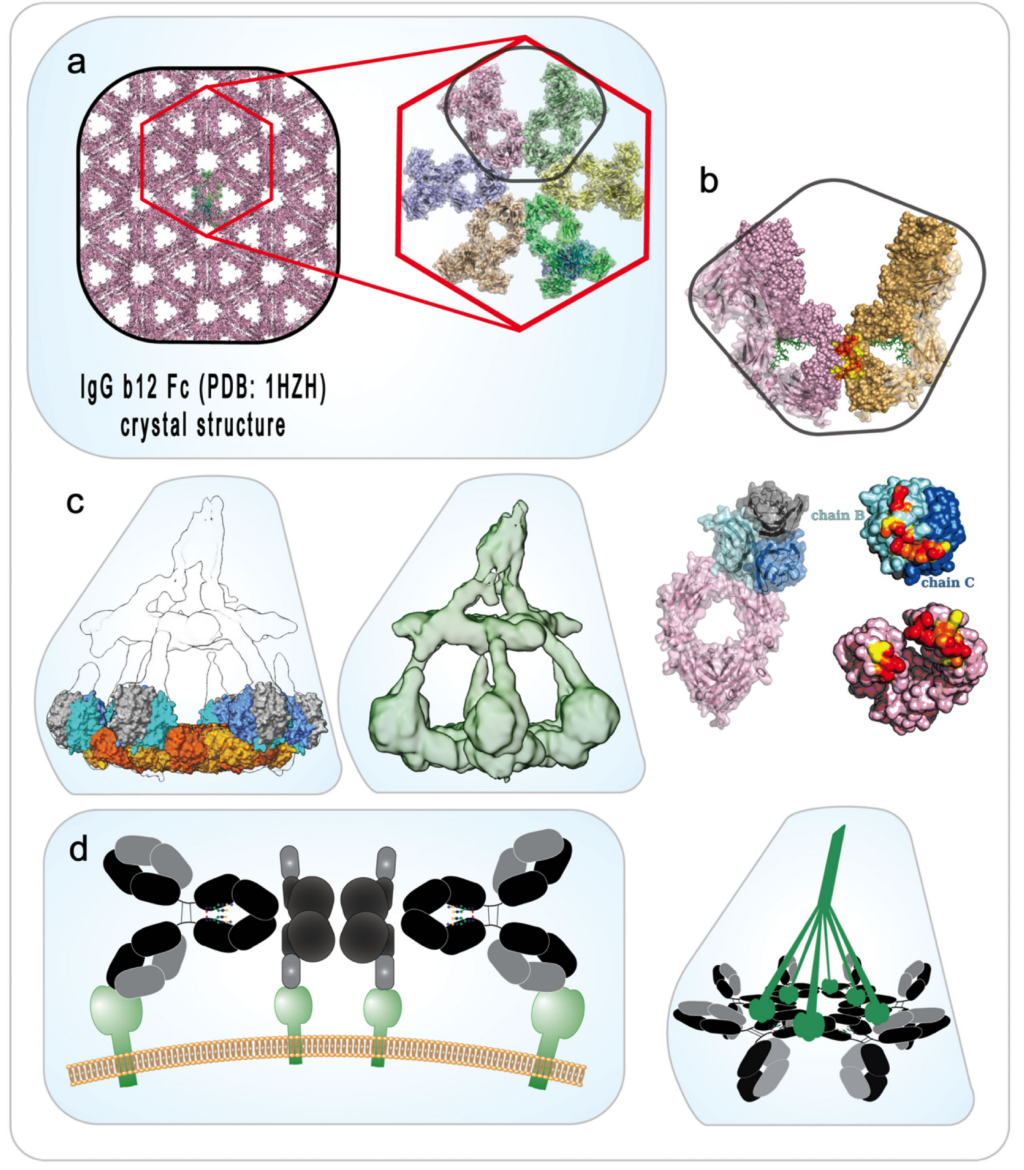
Structural determination of the classical complement activation complex provides mechanistic framework. (a) The repeating hexagonal lattice structure evident in crystals of an early structure of a whole IgG1 molecule (78) provided evidence that antibodies might self-associate. Diebolder and colleagues leveraged this clue to experimentally demonstrate that antibody-mediated complement activation is a function of (b) Fc:Fc association, in addition to the ability of gC1q to bind to the CH2–hinge region (25). (c) The 3D reconstruction of soluble C1–IgG16 complex (right panel; EMD-4232) as determined using single particle cryo-electron microscopy (80). The left panel illustrates a model of the C1–IgG16 complex with the IgG Fc:gC1q structure (PDB 6FCZ) fit sixfold into the density C1–IgG16 tomograph. (d) Monovalent target engagement, in some cases, promotes more efficient C1q recruitment through both gC1q:Fc recognition site accessibility and stabilization by noncovalent interactions between unliganded Fab and gC1q. gC1q Globular head of the C1q molecule.

Moving beyond a simple model in which antibody density relates directly to AMEF, structural studies of IgG1 have firmly established its ability to hexamerize (25), an attribute hinted at by early structural studies of a full IgG1 molecule (Fig. 3a) (78). Fc-to-Fc contacts mediate the formation of an optimal hexameric recruitment platform for the C1 complex and activation of the classical complement cascade (Fig. 3b and c). Similarly, elegant studies have recently been reported for human IgG3, which demonstrated how the extended hinge domain of certain IgG3 allotypes not only allows for more densely-spaced Fab packing on target surfaces but also generates hexameric Fc platforms elevated approximately twice the distance from the surface as those of IgG1 – both a higher density and less steric hinderance contributing to a greater degree of C1q binding (79). Interestingly, the IgG3 hexamers caused subsequently bound C1 to adopt conformations associated with autoactivation, and uniquely, C4b was found to be deposited at the CH1–hinge interface with implications for cascade amplification.

This framework helps explain the functional heterogeneity observed by antibodies expected to be intrinsically capable of complement activation, and supports investigation into the spatial determinants of epitope- (31, 32, 77) and binding mode-dependent activity (81, 82). For example, an IgG1 HIV-1-specific mAb that is capable of interacting with C1q and initiating the complement cascade (1, 83) was nonetheless functionally null when assessed for complement-mediated lysis of infected primary T cells (30), Env surface-expressing cell lines, and cell-free virus (30, 83). It is hypothesized that this discrepancy stems from assay design considerations in which differential accounting for features, such as antigen expression levels, the presence of complement-restricting host factors, and 3D binding geometries impact whether or not AMEF activity is observed (Fig. 3d).

## COMPONENTS CONTRIBUTING TO AMEF

**Fig 4:**
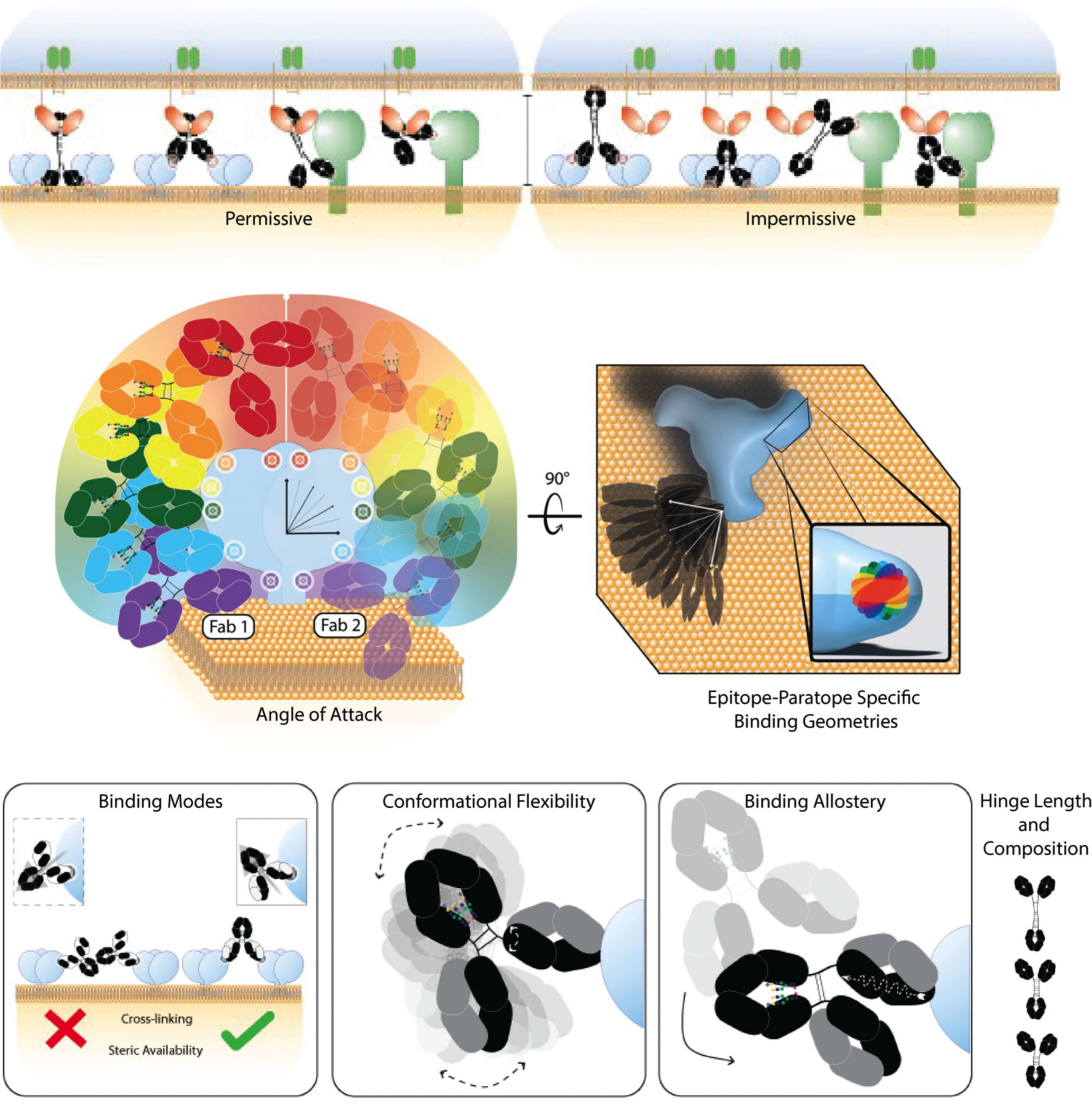
Spatial factors at antibody-mediated synapses may partially explain phenotypic heterogeneity observed within groups of similar antibodies. Top. Spatial consequences emerging from the interplay between antibody, effector, and target features at the antibody-mediated immune synapse influence the ability or degree to which cross-linking and activating/inhibitory signaling occurs. Center. Epitope location constrains ability of Fc and other Fab arm(s) to achieve FcR and avid binding, respectively. The asymmetric nature of the Fc makes it possible for binding by either Fab to result in distinct Fc orientation. Bottom. The epitope-paratope structure unique to every antibody-antigen pair, and malleable with antigen conformation or integrity, determines both the steric availability of the Fc and the ability for multivalent modes of binding. Antibody characteristics influencing the overall conformational flexibility affects the probability of antigen cross-linking and FcR interactions. Antigen binding-induced conformational changes may also play a role in regulating antibody-mediated effector functions, as does the flexibility and length of the antibody hinge.

In developing antibodies as therapeutic interventions, it can be helpful to understand how their structural features relate to their repertoire of functions. Conceptually modelled as a tripartite interaction between antibody, target, and effector, antibody activities are constrained by the parameters that influence the kinetics and binding modes of their individual interactions and the dynamics of the system (Fig. 1). In the context of drug development, the biophysical features of antibodies that influence diverse AMEF (6, 17, 72, 84–99) are tunable by format (e.g., isotype/subclass) selection and antibody engineering, while genetic/environmental variability and selective pressures are sources of effector and target heterogeneity (100) that are more difficult to control, but no less impactful. Studies of other ISs suggest that the integration of variable Fc steric availability and target antigen crosslinking likely translate to heterogeneous levels of cellular AMEF activities (Fig. 4a). It is also possible, however, that optimal Fc spatial geometries exist for effector recognition and IS formation and signaling (Fig. 4b) (101). Here, building from intrinsic features, we describe some of the factors affecting AMEF.

### Target antigen parameters

The nature of the target molecule(s) impact IC structure and AMEF. Whole or fragmented target antigen (Ag) can be found on the surfaces of fungal, bacterial, viral, and multicellular pathogens, pathogen-infected or malignant host cells, and as soluble protein. Beyond the presence or absence of immunostimulatory or inhibitory signals, the structural identity and local positioning of Ag plays a significant role in AMEF activity by constraining possible antibody engagement modes (e.g*.,* the ability to bind in a multivalent fashion). For example, sequence and structurally variable viral fusion proteins, such as HIV-1 Env, variably expose epitopes to mAbs, resulting in a range of sensitivities of infected cells to ADCC (35). The interplay of host and pathogen factors also influence conformational epitope exposure (102, 103). Additionally, the combination of an Ag’s oligomeric state (104), expression density (105), membrane-embedded diffusion profile (63), and spatial organization (75, 106) determine whether polyvalent complex formation is probable. Indeed, as an extreme example, target Ag density has been reported to influence whether mAbs exhibit infection enhancing or neutralizing activity *in vitro* (107).

### Classical antibody intrinsic factors

The most important antibody intrinsic parameters shaping AMEF are isotype (11, 108–110) and subclass (111–114). In addition to broad differences in their primary amino acid sequences, which define their cognate receptor-binding profiles, antibody isotypes and subclasses differ in the nature of target engagement due to differences in binding modes (81, 82), Fc valency (73), hinge composition and conformation (115–118), as well as overall conformational plasticity (Fig. 3b) (114, 119–121).

The N-linked Fc glycan arguably represents the second most impactful antibody parameter underlying AMEF. Afucosylation of the conserved Fc glycan core results in an increased affinity toward FcγRIIIa and often, but not always, a commensurate enhancement in ADCC activity (122–126). More modest impacts on AMEF are known to exist between certain glycoforms composed of terminal galactose moieties and AMEFs, such as ADCC (124) and ADCML (127). In recent years, a structural basis for the observations linking certain terminal galactosylated species with classical complement activation have been determined to possibly result from impacts on Fc-to-Fc interactions, which influence formation of the prototypical complement activation complex (128, 129), as opposed to Fc-to-FcR. These observations have been leveraged in the design and trial of over 25 – and regulatory approval of at least three – glycoengineered therapeutic mAbs (130). Cumulatively, differences in isotype and subclass combine with variation in Fc glycosylation (122, 123) to place constraints on Fc spatial orientation (131, 132), modulate receptor engagement (133, 134), and thereby influence the nature of higher-order complex formation (25, 128, 135).

The final major antibody-intrinsic determinants of AMEFs are Ag recognition properties. Beyond the Ag- or epitope-associated factors, such as copy number, post-translational modifications, and others described previously, the antibody’s intrinsic affinity for Ag, and its component binding association and dissociation rate constants play a role in AMEF (62, 136–138). Complicating interpretation of monovalent affinities measured between antibody Fab domains and Ag, or Fc domains and FcR, a number of studies have generated support for an allosteric model, in which binding at each functional end of the antibody is not independent, but is instead linked and can influence distal interaction kinetics and equilibrium-binding affinities (29, 139, 140), as described further below.

### Effector biology

Effector cells/proteins represent the final of the three components involved in AMEF. Expression levels, bioavailability (141, 142), and genotype (85) of the 30-plus protein components of the complement system vary, and some have been linked to the therapeutic profile of antibodies (85). Parameters that influence cellular AMEF are even more varied and complex (143), including variation in FcR-intrinsic biophysical features (e.g., N-linked glycan and genetic variation), FcR copy number, membrane composition and membrane mobility, cytoskeletal remodeling, resting and post-engagement FcR clustering, and levels of regulatory and downstream signaling factors. Multiple types and polymorphic variants of FcRs exist, each with unique antibody isotype and subclass affinities/specificities (144–146), and are present on different effectors in distinct combinations (147). Numerous differences exist both among humans and between humans and animal models commonly used in preclinical studies (146, 148–150). Furthermore, like other intracellular components of the relevant pathways, the types of FcR and their relative and absolute surface expression levels, diffusion, and signaling (151–153) patterns are not fixed, but vary dynamically in response to local cues.

## COMPONENTS IN COMBINATION

While considering effector, antibody, and antigen as discreet, modular components has had great advantages in terms of simplicity, there are many examples of emergent behavior in which either the inherent complexity of these biological systems or unappreciated biology have led to surprising observations. In the following sections, we highlight further some examples of this growing body of work.

### Complex interactions in antibody-mediated effector functions

The effects of classical complement activation and opsonization (6, 72, 85), antibody-dependent cellular phagocytosis (ADCP) by myeloid cells (6, 17, 86, 87), NK cell activation (154, 155), and ADCC (88–90) are antibody-mediated effector mechanisms that have been a particular focus of study. Additional functions elicited by these and other immune cell subsets have been observed. For example, neutrophils mediate phagocytosis (ADNP) and other activities, such as respiratory burst (91) and neutrophil extracellular trap expression (92), through both IgG-FcγR and IgA-FcαR ligation. Additional antibody-mediated processes include immunomodulation by exchange of membrane components from coincident immune cells in a process known as trogocytosis (93–96), degranulation (156), physical target cell destruction in a process termed trogoptosis or antibody-dependent fragmentation (97, 98), and the secretion of a range of functionally potent cytokines and chemokines by various immune cells (99).

Antibodies can activate multiple mechanisms of action simultaneously, further complicating understanding and modeling of the system. Signaling crosstalk or steric factors may amplify or interfere with diverse AMEF. For example, complement opsonins can be recognized by complement receptors expressed on hematopoietic cells and mediate immune adherence and trafficking, adaptive immune education, and complement-dependent cellular cytotoxicity and phagocytosis (157, 158). While simultaneous mAb and complement fragment opsonization has indeed been shown to inhibit ADCC (159), virus internalization by ADCP was recently reported to be augmented when a hexamer-promoting Fc variant was tested in the presence of complement (160).

### Integration of Fc receptor signaling

Antibody isotypes, subclasses, and allotypic variants intrinsically possess an interaction profile with diverse FcγRs, and those FcγRs, in turn, are linked to intracellular domains that transduce either activating or inhibitory signals. The relative simultaneous contribution of these opposing signals influences the level of resultant activity. This fact is important in antibody engineering efforts because altering the affinity for one FcγR often impacts other interactions with the highly homologous FcγRs. In one example, an α-EGFR mAb Fc-engineered to better facilitate FcγRIIIa-mediated NK ADCC activity showed increased inhibition of neutrophil ADCC mediated by FcγRIIIb. Neutrophil activation could be restored by introducing Fc mutations that also improved recognition of FcγRIIa (161).

Effector cell activation and functional outcomes result from the types, relative levels of activating versus inhibitory FcRs, and the degree of FcR crosslinking and resultant intracellular signaling pathway induction, which are influenced by the cell type (162), education (154), and polarization status shaping and shaped by the inflammatory micro-environmental milieu. Missing from this picture is a nuanced view behind the term “FcR crosslinking.” Considering the role of supramolecular structures and dynamics on T cell IS formation and phenotypes, there could be a connection between the detailed structural characteristics of specific FcR–Fc interactions, influenced by the spatial constraints of Ag-binding, and the signaling strength of the resulting FcR-crosslinked supramolecular structures. Furthermore, target- and effector cell-specific membrane factors, architecture, and dynamics may also contribute to nuanced and variable function kind and degree (e.g., phagocytosis vs trogoptosis).

### Spatial effects

The prevailing model of cellular AMEF holds that effector sensing and subsequent activation is mediated by avidity-based Fc-FcR clustering. In addition to this process, in which multiple low affinity interactions combine to result in high effective affinity, it has become clear that additional spatial factors and interactions can play important roles. In principle, differing epitopes range spatially from the base of the Ag, proximal to the membrane, to the Ag’s apex, distal to the membrane (Fig. 4b). And because the Fc is asymmetric, target engagement by one Fab does not result in equivalent 3D orientation of the Fc following binding by the other Fab. Additionally, for any given epitope, the angles at which bound antibodies interact can vary, and as a result, the spatial orientation of the Fc falls on a continuum from mostly obscured to easily available to effectors (Fig. 4b). Even for antibodies that recognize the same or overlapping epitopes, this can result in divergent AMEF activities. In a striking example of this point, Acharya et al. described the structural basis underpinning a 75-fold difference in ADCC potency between two competing HIV-1-specific antibodies, N5-i5 (high ADCC) and 2.2c (low ADCC), that recognize overlapping epitopes on monomeric Ag with equivalent intrinsic affinity (163). On the surface of target cells, however, saturation levels of N5-i5 were twice that of 2.2c, and it was determined that N5-i5 more efficiently formed complexes of cross-linked Ag. Structural work revealed that when bound to Ag, the Fab domains of the two antibodies were rotated ~180° in relation to one another, which was predicted to orient the Fc domain of 2.2c towards the viral membrane, and less available to FcR. To test the hypothesis that the Fc angle impacted ADCC activity of 2.2c, the investigators generated chimeras with swapped VH and VL domains such that once Ag-bound, their Fc domains would be spatially inverted (Fig. 4c). This experiment yielded a spatially chimeric antibody with native binding affinity and sevenfold improved ADCC activity. This work elegantly demonstrated that the structural consequences of specific epitope-paratope pairs influence the spatial orientation of Ag-bound antibody, affecting the potential of the unbound Fab to mediate multivalent Ag-Ab complex formation and of the Fc domain to engage effectors (Fig. 4c).

The impact of monovalent versus bivalent IgG binding on complement activity is instructive of how spatial constraints imposed by Ag binding influence effector engagement. Diebolder and colleagues generated two bsAbs targeting distinct Ags that each had one target-specific arm and one irrelevant Fab, and found that in one of the two cases, the monovalent bsAb elicited greater complement lysis than the parental, bivalent mAb (25). This result demonstrated that on an Ab-Ag basis, monovalent binding could be more impactful than avid, high apparent affinity binding. Within the context of the authors’ complement activation model, in which Fc-to-Fc association forms the basis for optimal complement activation, monovalent binding theoretically led to greater complement recruitment by allowing for greater spatial degrees of freedom to support efficient oligomerization. This is also a convenient explanation for the observation that polyclonal or combinations of mAbs recognizing distinct epitopes are often more potent complement-activators than mAbs (164–166). In other cases, however, perhaps where high surface Ag densities provide an important offset for low monovalent affinity, bivalent binding correlates with higher complement dependent activity (81, 82).

Beyond intramolecular structural changes to IgG that impact activity, intermolecular factors also play an important role. Refined structural insights have emerged from Fc engineering approaches that manipulate formation of a hexameric IgG (25, 167), and the resultant model has served as the basis for interpreting the functional consequences of immune complex superassemblies formed by rituximab and CD20 dimers (168, 169), and of epitope-specific differences in the structures of two Ag-bound Type I α-CD20 mAbs, rituximab and ofatumumab (163). In addition to enriching a mechanistic understanding of approved and candidate clinical mAb function, this structural model of antibody-mediated complement activation has enabled manipulation of *in vivo* activity (160, 164). For example, IgG1 Fc mutants (e.g., E430G, E345K) that enable the potentiation of antibody-mediated complement activation in a diverse range of therapeutic antibodies were identified from a panel of modified Fc:Fc interface residues. The E430G (“EG”) mutant has since been determined to be of translational interest (170), having shown promising preclinical activity in combination oncotherapeutic settings (166, 171) and HIV (172), and is currently under clinical evaluation for the treatment of relapsed or refractory hematologic malignancies (ClinicalTrials.gov Identifier: NCT04824794).

### Allostery

There is a growing base of evidence that points to the ability of sites outside of complementarity determining regions (CDRs) to significantly modify antibody structure and thereby activity. Intriguingly, the composition of the variable domain framework region has been implicated in affecting antigen binding modes and activities, sometimes in dramatic ways (173). For example, *in silico-*guided manipulation of the VH–VL interface has been employed to tune affinity and stability (174), and single point mutations have altered CDR loop conformational diversity and interdomain orientations (175) – in one case imparting novel inhibitory activity of direct binding by potentiating an intra-antibody VH–VH interaction (176). These results are reminiscent of the well-established but paradoxical impacts of antibody hinge conformation on activity, which include IgG2 isomers that display differences in their ability to undergo FcRn-mediated transcytosis (177), induce apoptosis (178), and possess super-agonistic properties (116).

While essential to activation, associative interaction is not the only proposed mechanism contributing to Ab-effector ligand sensing. Some studies have suggested that the structural composition of the Fc domain affects the Ag binding properties of the Fab (179). Still others have suggested that the act of Ag binding prompts conformational changes through to the Fc domain, affecting or improving its ability to engage FcR (Fig. 4e) (29, 140, 180, 181). Many questions around antibody allostery remain open and are hotly debated, but what is clear is that there exist structural and spatial nuances that emerge from the biophysical nature of the antibody.

Investigation of the functional determinants of agonistic cancer immunotherapy mAbs targeting co-stimulatory molecules of the tumor necrosis factor receptor superfamily has revealed a strong interdependent relationship between epitope and isotype (33, 34, 182). For α-CD27 agonists targeting membrane-distal and external facing epitopes tended to produce stronger activity, while poorer agonists could be improved by introducing mutations into the IgG1 Fc that improved affinity to FcγRIIb, or by reformatting into an IgG2 backbone (34). In fact, the characteristic disulfide bonding of the IgG2 hinge was shown to be indispensable for conversion of an α-CD40 antagonist to an FcγR-independent super-agonist (109).

## TOWARDS A FULLY INTEGRATED MODEL OF AMEF

The development of antibody interventions requires *in vitro* functional screening and low-throughput *in vivo* experimentation to establish safety and efficacy. Efforts to model complex multi-molecular systems derive from investigation of Ab-target-effector systems, and extend well beyond the realm of antibody complexes in their application (20, 183–186). New insights afforded by structural studies of the B cell receptor (BCR) (187–189) offer the potential to expand understanding of how some of the same factors that influence AMEF may play a role in BCR stimulation. Efforts to build and improve predictive models of *in vivo* antibody behavior are clearly underway. As *in silico* structural modeling methods (21, 22, 24) continue to make progress towards accurate and reliable predictions of higher-order complexes, more sophisticated field applications will likely emerge. We anticipate that more detailed understanding of how a given antibody will engage immune effectors *in vivo* will evolve from the current picture of FcR-binding/kinetic profiles and *in vitro* assays coupled to structural insights into the spatial implications of Ab–target complex formation as they relate to a range of effector cells.
